# 5-(1-Cyclo­hexen-1-yl)-3-(4-methoxy­phen­yl)isoxazole

**DOI:** 10.1107/S1600536809010903

**Published:** 2009-03-28

**Authors:** Gabriel Vallejos, Margarita Gutierrez, Luis Astudillo, Iván Brito, Alejandro Cárdenas

**Affiliations:** a Laboratorio de Biorgánica, Instituto de Química, Facultad de Ciencias, Universidad Austral de Chile, Casilla 567, Isla Teja S/N, Valdivia, Chile; b Laboratorio de Síntesis Orgánica, Instituto de Química de Recursos Naturales, Universidad de Talca, Casilla 747, Talca, Chile; c Departamento de Química, Facultad de Ciencias Básicas, Universidad de Antofagasta, Casilla 170, Antofagasta, Chile; d Departamento de Física, Facultad de Ciencias Básicas, Universidad de Antofagasta, Casilla 170, Antofagasta, Chile

## Abstract

In the title compound, C_16_H_17_NO_2_, the isoxazole ring makes a dihedral angle of 14.81 (13)° with the 4-methoxy­phenyl ring. Two atoms of the cyclo­hexene ring are disordered over two almost equally occupied positions [0.526 (13)/0.474 (13)]. The mol­ecular structure features a short intra­molecular C—H⋯O contact.

## Related literature

For background to isoxazoles, see: Melo (2005 ▶). For their biological activities, see: Narlawar *et al.* (2008 ▶); Patrick *et al.* (2007 ▶); Taldone *et al.* (2008 ▶); Rizzi *et al.* (2008 ▶); Velaparthi *et al.* (2008 ▶). For synthetic details, see: Hansen *et al.* (2005 ▶).
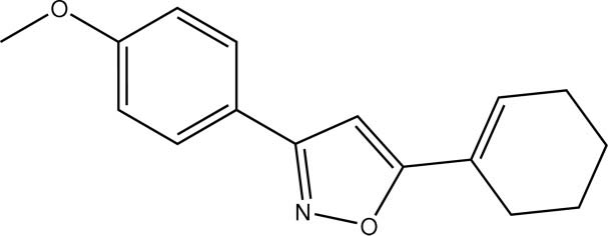


## Experimental

### 

#### Crystal data

C_16_H_17_NO_2_
*M* *_r_* = 255.31
Triclinic, 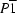

*a* = 5.8690 (11) Å
*b* = 10.9646 (19) Å
*c* = 11.481 (5) Å
α = 77.889 (2)°
β = 75.728 (5)°
γ = 80.262 (9)°
*V* = 694.7 (4) Å^3^
*Z* = 2
Mo *K*α radiation
μ = 0.08 mm^−1^
*T* = 295 K
0.20 × 0.16 × 0.10 mm

#### Data collection

Nonius KappaCCD area-detector diffractometer
Absorption correction: none
4217 measured reflections
2467 independent reflections
2023 reflections with *I* > 2σ(*I*)
*R*_int_ = 0.076

#### Refinement

*R*[*F* ^2^ > 2σ(*F* ^2^)] = 0.055
*wR*(*F* ^2^) = 0.135
*S* = 1.14
2467 reflections
192 parameters
H-atom parameters constrained
Δρ_max_ = 0.14 e Å^−3^
Δρ_min_ = −0.20 e Å^−3^

### 

Data collection: *COLLECT* (Nonius, 2000 ▶); cell refinement: *DENZO-SMN* (Otwinowski & Minor, 1997 ▶); data reduction: *DENZO-SMN*; program(s) used to solve structure: *SIR97* (Altomare *et al.*, 1999 ▶); program(s) used to refine structure: *SHELXL97* (Sheldrick, 2008 ▶); molecular graphics: *ORTEP-3 for Windows* (Farrugia, 1997 ▶) and *PLATON* (Spek, 2009 ▶); software used to prepare material for publication: *WinGX* (Farrugia, 1999 ▶).

## Supplementary Material

Crystal structure: contains datablocks global, I. DOI: 10.1107/S1600536809010903/bt2910sup1.cif

Structure factors: contains datablocks I. DOI: 10.1107/S1600536809010903/bt2910Isup2.hkl

Additional supplementary materials:  crystallographic information; 3D view; checkCIF report

## Figures and Tables

**Table 1:** Hydrogen-bond geometry (Å, °)

*D*—H⋯*A*	*D*—H	H⋯*A*	*D*⋯*A*	*D*—H⋯*A*
C6—H6⋯O1	0.93	2.48	2.811 (3)	101

**Fig. 1.**
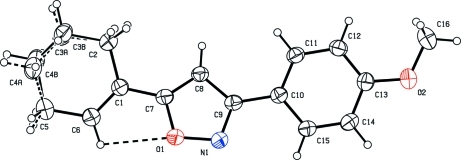
A view of the molecular strucuture of (I) with 30% probability displacement ellipsoids. H atoms are drawn as small spheres of arbitrary radii and intramolecular hydrogen bond is indicated by dotted lines. Atoms C3, C4 and hydrogen atoms bonded to C2 and C5 are severely disordered.

**Fig. 2.**
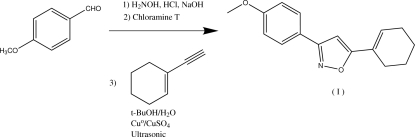
The formation of the title compound.

## supplementary crystallographic information

### Comment

Isoxazoles are molecules of great interest in chemistry because many natural products have been synthesized starting from these ones (Melo, 2005), and also because these compounds exhibit diverse biological activities [*i.e.*antiprotozoalactivities (Patrick *et al.*, 2007), Hsp90 superchaperone complex inhibitors (Taldone *et al.*, 2008), tau aggregation inhibitors for treatment of Alzheimer's disease (Narlawar *et al.*, 2008) *Mycobacteriumtuberculosis* pantothenate synthetase inhibitors (Velaparthi *et al.*, 2008), and neuronal nicotinic acetylcholine receptors agonist (Rizzi *et al.*, 2008)]. This compound was evaluated against acetilcholinesterase (AChE) enzyme, it showed moderate inhibitory activity toward AChE, with a IC_50 _of 2.16 m*M*. For these reasons, the synthesis and structure of isoxazole is still of great interest. We report here the crystal structure of the title compound, Fig.1. The planar isoxazole makes a dihedral angle of 14.85 (13)° with the 4-methoxyphenyl ring and 25.1 (3)° and 14.1 (3)° with the  cyclohexene groups, respectively. The molecular structure is stabilized by one intramolecular C—H··· O hydrogen bond, Table 1.

### Experimental

Melting points were recorded on an Electrothermal 9100 instrument and are uncorrected; IR spectra were obtained on a Nicolet Nexus 470-FTIR spectrometer as potassium bromide pellets and are reported in wavenumbers (cm^-1^). ^1^H and ^13^C NMR spectra were measured on a Bruker AM-400 spectrometer (400 MHz), using CDCl_3_ assolvent. TMS was used as an internal standard. Chemical shifts (*d*) and *J* values are reported in p.p.m. and Hz, respectively. Reaction progress was monitored by means of thin-layer chromatography using Merck Kieselgel 60 (230–240 mesh). All reagents were purchased from Merck, Sigma and Aldrich Chemical Co. and used without further purification. Solvents were dried and distilled prior to use.

5-(1-cyclohexen-1-yl)-3-(4-methoxyphenyl)isoxazole was obtained using the method described by Hansen *et al.* (2005), starting from 4-methoxybenzaldehyde (2.72 g, 20 mmol), hydroxylamine hydrochloride (1.46 g, 21 mmol), chloramine-T trihydrate (5.9 g, 21 mmol) and 1-Ethynylcyclohexene (2.23 g,21 mmol) (See Fig. 2), giving off-white solid; mp 76–78 °C, yield 93%. Yellow block-shaped crystals of (I) suitable for X-ray analysis were grown from a hexane/EtOAc solution (1:1 *v*/*v*) at 298 K over a period of a few days. RMN-^1^H(CDCl_3_, 400 MHz): d 7,74 (d, *J*= 8.9 Hz, 2H); 6,96 (d, *J*= 8.9 Hz, 2H); 6,64 (m, 1H); 6,32 (s,1*H*); 3,85 (s, 1H); 2,37 (m, 2H); 2,26 (m, 2H); 1,77 (m, 2H); 1,69 (m, 2H). RMN-^13^C(CDCl_3_, 100 MHz): d 171.35, 161.99,160.83, 129.98, 128.07, 125.40, 121.98, 114.21, 95.93, 55.31, 25.39, 25.20,22.08, 21.70. F T–IR (KBr pellet, cm^-1^): 3851, 2935, 1652, 1525, 1430, 1248, 1177, 1030, 919.

### Refinement

All H atoms were positioned geometrically with C—H = 0.93–0.97 Å and refined as riding model, with *U*_iso_(H) = 1.2 or 1.5 times *U*_eq_(C) for aromatic or methyl H atoms respectively. Atoms C3, C4 and hydrogen atoms bonded to C2 and C5 are severely disordered. They were modelled using a split model with refined population parameters [C3A/C3B = 0.474 (13)/0.526 (13); C4A/C4B = 0.474 (13)/0.526 (13); H2A/H2C= H2B/H2D = 0.474 (13)/0.526 (13); H5A/H5C=H5B/H5D = 0.474 (13)/0.526 (13)].

### Crystal data

**Table:**

C_16_H_17_NO_2_	*Z* = 2
*M**_r_* = 255.31	*F*(000) = 272
Triclinic, *P*1	*D*_x_ = 1.221 Mg m^−^^3^
Hall symbol: -P 1	Mo *K*α radiation, λ = 0.71073 Å
*a* = 5.8690 (11) Å	Cell parameters from 2123 reflections
*b* = 10.9646 (19) Å	θ = 1.9–24.4°
*c* = 11.481 (5) Å	µ = 0.08 mm^−^^1^
α = 77.889 (2)°	*T* = 295 K
β = 75.728 (5)°	Block, yellow
γ = 80.262 (9)°	0.20 × 0.16 × 0.10 mm
*V* = 694.7 (4) Å^3^	

### Data collection

**Table:**

Nonius KappaCCD area-detector diffractometer	2023 reflections with *I* > 2σ(*I*)
Radiation source: fine-focus sealed tube	*R*_int_ = 0.076
graphite	θ_max_ = 25.2°, θ_min_ = 3.6°
φ scans, and ω scans with κ offsets	*h* = 0→7
2467 measured reflections	*k* = −12→13
2467 independent reflections	*l* = −12→13

### Refinement

**Table:**

Refinement on *F*^2^	Primary atom site location: structure-invariant direct methods
Least-squares matrix: full	Secondary atom site location: difference Fourier map
*R*[*F*^2^ > 2σ(*F*^2^)] = 0.055	Hydrogen site location: inferred from neighbouring sites
*wR*(*F*^2^) = 0.135	H-atom parameters constrained
*S* = 1.14	*w* = 1/[σ^2^(*F*_o_^2^) + (0.0545*P*)^2^ + 0.128*P*] where *P* = (*F*_o_^2^ + 2*F*_c_^2^)/3
2467 reflections	(Δ/σ)_max_ < 0.001
192 parameters	Δρ_max_ = 0.14 e Å^−^^3^
0 restraints	Δρ_min_ = −0.20 e Å^−^^3^

### Special details

**Table:**

Geometry. All s.u.'s (except the s.u. in the dihedral angle between two l.s. planes) are estimated using the full covariance matrix. The cell s.u.'s are taken into account individually in the estimation of s.u.'s in distances, angles and torsion angles; correlations between s.u.'s in cell parameters are only used when they are defined by crystal symmetry. An approximate (isotropic) treatment of cell s.u.'s is used for estimating s.u.'s involving l.s. planes.
Refinement. Refinement of *F*^2^ against ALL reflections. The weighted *R*-factor *wR* and goodness of fit *S* are based on *F*^2^, conventional *R*-factors *R* are based on *F*, with *F* set to zero for negative *F*^2^. The threshold expression of *F*^2^ > 2σ(*F*^2^) is used only for calculating *R*-factors(gt) *etc*. and is not relevant to the choice of reflections for refinement. *R*-factors based on *F*^2^ are statistically about twice as large as those based on *F*, and *R*- factors based on ALL data will be even larger.

### Fractional atomic coordinates and isotropic or equivalent isotropic displacement parameters (Å^2^)

**Table:**

	*x*	*y*	*z*	*U*_iso_*/*U*_eq_	Occ. (<1)
O1	0.3554 (2)	0.17491 (13)	−0.04016 (12)	0.0640 (4)	
O2	−0.2921 (2)	0.60615 (14)	0.41851 (13)	0.0715 (4)	
N1	0.3139 (3)	0.25243 (16)	0.04881 (15)	0.0628 (5)	
C1	0.1547 (3)	0.10166 (16)	−0.16684 (15)	0.0495 (4)	
C2	−0.0473 (4)	0.1345 (2)	−0.2317 (2)	0.0690 (6)	
H2A	−0.0272	0.2116	−0.2907	0.083*	0.474 (13)
H2B	−0.1962	0.1473	−0.1734	0.083*	0.474 (13)
H2C	−0.0855	0.2253	−0.2471	0.083*	0.526 (13)
H2D	−0.1854	0.0998	−0.178	0.083*	0.526 (13)
C3A	−0.0493 (12)	0.0236 (11)	−0.2981 (9)	0.071 (2)	0.474 (13)
H3A1	−0.1043	−0.0477	−0.238	0.106*	0.474 (13)
H3A2	−0.1588	0.0494	−0.3523	0.106*	0.474 (13)
C4A	0.1966 (18)	−0.0159 (11)	−0.3718 (9)	0.081 (2)	0.474 (13)
H4A1	0.1885	−0.0773	−0.4199	0.121*	0.474 (13)
H4A2	0.2607	0.0564	−0.4266	0.121*	0.474 (13)
C5	0.3446 (4)	−0.0697 (2)	−0.2859 (2)	0.0758 (6)	
H5A	0.5079	−0.0816	−0.3303	0.091*	0.474 (13)
H5B	0.2998	−0.1517	−0.245	0.091*	0.474 (13)
H5C	0.3818	−0.1576	−0.252	0.091*	0.526 (13)
H5D	0.4711	−0.0468	−0.3556	0.091*	0.526 (13)
C6	0.3280 (3)	0.00944 (18)	−0.19162 (18)	0.0607 (5)	
H6	0.4466	−0.007	−0.1476	0.073*	
C7	0.1488 (3)	0.17676 (16)	−0.07484 (15)	0.0483 (4)	
C8	−0.0224 (3)	0.25240 (16)	−0.01282 (15)	0.0494 (4)	
H8	−0.1798	0.2716	−0.0194	0.059*	
C9	0.0881 (3)	0.29654 (16)	0.06460 (16)	0.0475 (4)	
C10	−0.0167 (3)	0.37884 (16)	0.15521 (15)	0.0470 (4)	
C11	−0.2372 (3)	0.44858 (17)	0.15767 (16)	0.0534 (5)	
H11	−0.3215	0.4431	0.1006	0.064*	
C12	−0.3358 (3)	0.52620 (17)	0.24271 (17)	0.0550 (5)	
H12	−0.4838	0.5726	0.242	0.066*	
C13	−0.2127 (3)	0.53436 (17)	0.32878 (17)	0.0539 (5)	
C14	0.0074 (3)	0.4641 (2)	0.32840 (19)	0.0650 (5)	
H14	0.0903	0.4686	0.3864	0.078*	
C15	0.1035 (3)	0.38833 (19)	0.24361 (18)	0.0605 (5)	
H15	0.2516	0.3422	0.2446	0.073*	
C16	−0.5130 (4)	0.6839 (2)	0.4212 (2)	0.0756 (6)	
H16A	−0.5046	0.7435	0.3464	0.113*	
H16B	−0.5476	0.7279	0.4887	0.113*	
H16C	−0.6359	0.6327	0.4303	0.113*	
C3B	0.0006 (13)	0.0880 (9)	−0.3512 (8)	0.0726 (19)	0.526 (13)
H3B1	0.1138	0.1355	−0.4124	0.087*	0.526 (13)
H3B2	−0.1448	0.0981	−0.3799	0.087*	0.526 (13)
C4B	0.0995 (17)	−0.0499 (7)	−0.3290 (8)	0.0731 (19)	0.526 (13)
H4B1	0.1244	−0.0833	−0.4036	0.11*	0.526 (13)
H4B2	−0.0147	−0.0961	−0.267	0.11*	0.526 (13)

### Atomic displacement parameters (Å^2^)

**Table:**

	*U*^11^	*U*^22^	*U*^33^	*U*^12^	*U*^13^	*U*^23^
O1	0.0476 (7)	0.0832 (9)	0.0673 (9)	0.0055 (6)	−0.0185 (6)	−0.0305 (7)
O2	0.0740 (9)	0.0768 (9)	0.0685 (9)	0.0023 (7)	−0.0157 (7)	−0.0325 (8)
N1	0.0514 (9)	0.0822 (11)	0.0622 (10)	0.0027 (8)	−0.0193 (7)	−0.0297 (9)
C1	0.0466 (10)	0.0553 (10)	0.0451 (10)	−0.0090 (8)	−0.0065 (7)	−0.0073 (8)
C2	0.0610 (12)	0.0819 (14)	0.0743 (14)	−0.0005 (10)	−0.0252 (10)	−0.0304 (12)
C3A	0.062 (3)	0.087 (6)	0.075 (5)	−0.004 (3)	−0.027 (3)	−0.029 (4)
C4A	0.079 (5)	0.106 (6)	0.063 (5)	−0.008 (4)	−0.010 (4)	−0.039 (4)
C5	0.0782 (14)	0.0745 (14)	0.0755 (15)	0.0014 (11)	−0.0122 (12)	−0.0294 (12)
C6	0.0594 (11)	0.0648 (12)	0.0584 (12)	−0.0014 (9)	−0.0147 (9)	−0.0146 (10)
C7	0.0430 (9)	0.0559 (10)	0.0456 (10)	−0.0077 (7)	−0.0113 (7)	−0.0045 (8)
C8	0.0406 (9)	0.0601 (11)	0.0484 (10)	−0.0046 (7)	−0.0113 (7)	−0.0111 (8)
C9	0.0432 (9)	0.0525 (10)	0.0451 (10)	−0.0071 (7)	−0.0110 (7)	−0.0023 (8)
C10	0.0439 (9)	0.0529 (10)	0.0437 (10)	−0.0100 (7)	−0.0086 (7)	−0.0055 (8)
C11	0.0510 (10)	0.0624 (11)	0.0494 (11)	−0.0056 (8)	−0.0173 (8)	−0.0090 (9)
C12	0.0481 (10)	0.0588 (11)	0.0555 (11)	0.0009 (8)	−0.0131 (8)	−0.0084 (9)
C13	0.0568 (11)	0.0542 (11)	0.0501 (11)	−0.0103 (8)	−0.0083 (8)	−0.0092 (9)
C14	0.0571 (11)	0.0840 (14)	0.0632 (13)	−0.0055 (10)	−0.0233 (9)	−0.0242 (11)
C15	0.0470 (10)	0.0772 (13)	0.0627 (13)	0.0006 (9)	−0.0188 (9)	−0.0227 (10)
C16	0.0791 (14)	0.0645 (13)	0.0747 (15)	0.0025 (11)	−0.0032 (11)	−0.0189 (11)
C3B	0.090 (4)	0.072 (4)	0.066 (4)	−0.002 (3)	−0.035 (3)	−0.019 (3)
C4B	0.087 (5)	0.072 (4)	0.067 (4)	−0.003 (3)	−0.020 (4)	−0.029 (3)

### Geometric parameters (Å, °)

**Table:**

O1—C7	1.362 (2)	C5—H5D	0.97
O1—N1	1.413 (2)	C6—H6	0.93
O2—C13	1.370 (2)	C7—C8	1.348 (2)
O2—C16	1.424 (2)	C8—C9	1.420 (2)
N1—C9	1.313 (2)	C8—H8	0.93
C1—C6	1.330 (2)	C9—C10	1.472 (2)
C1—C7	1.460 (3)	C10—C11	1.384 (2)
C1—C2	1.506 (3)	C10—C15	1.400 (3)
C2—C3B	1.509 (6)	C11—C12	1.384 (2)
C2—C3A	1.569 (7)	C11—H11	0.93
C2—H2A	0.97	C12—C13	1.385 (3)
C2—H2B	0.97	C12—H12	0.93
C2—H2C	0.97	C13—C14	1.387 (3)
C2—H2D	0.97	C14—C15	1.366 (3)
C3A—C4A	1.525 (13)	C14—H14	0.93
C3A—H3A1	0.97	C15—H15	0.93
C3A—H3A2	0.97	C16—H16A	0.96
C4A—C5	1.442 (9)	C16—H16B	0.96
C4A—H4A1	0.97	C16—H16C	0.96
C4A—H4A2	0.97	C3B—C4B	1.517 (12)
C5—C6	1.499 (3)	C3B—H3B1	0.97
C5—C4B	1.601 (8)	C3B—H3B2	0.97
C5—H5A	0.97	C4B—H4B1	0.97
C5—H5B	0.97	C4B—H4B2	0.97
C5—H5C	0.97		
			
C7—O1—N1	108.69 (13)	C4B—C5—H5D	109.6
C13—O2—C16	118.24 (16)	H5B—C5—H5D	130.2
C9—N1—O1	105.65 (13)	H5C—C5—H5D	108.1
C6—C1—C7	121.93 (16)	C1—C6—C5	124.23 (19)
C6—C1—C2	121.99 (18)	C1—C6—H6	117.9
C7—C1—C2	116.08 (15)	C5—C6—H6	117.9
C1—C2—C3B	114.7 (3)	C8—C7—O1	108.99 (15)
C1—C2—C3A	108.3 (3)	C8—C7—C1	133.87 (16)
C1—C2—H2A	110	O1—C7—C1	117.14 (15)
C3B—C2—H2A	78	C7—C8—C9	105.51 (15)
C3A—C2—H2A	110	C7—C8—H8	127.2
C1—C2—H2B	110	C9—C8—H8	127.2
C3B—C2—H2B	129.1	N1—C9—C8	111.14 (16)
C3A—C2—H2B	110	N1—C9—C10	119.80 (15)
H2A—C2—H2B	108.4	C8—C9—C10	129.05 (15)
C1—C2—H2C	108.6	C11—C10—C15	117.38 (17)
C3B—C2—H2C	108.6	C11—C10—C9	121.84 (15)
C3A—C2—H2C	136.3	C15—C10—C9	120.78 (16)
H2B—C2—H2C	77.8	C12—C11—C10	121.89 (16)
C1—C2—H2D	108.6	C12—C11—H11	119.1
C3B—C2—H2D	108.6	C10—C11—H11	119.1
C3A—C2—H2D	81.5	C11—C12—C13	119.56 (16)
H2A—C2—H2D	133	C11—C12—H12	120.2
H2C—C2—H2D	107.6	C13—C12—H12	120.2
C4A—C3A—C2	111.4 (8)	O2—C13—C12	125.20 (17)
C4A—C3A—H3A1	109.3	O2—C13—C14	115.51 (16)
C2—C3A—H3A1	109.3	C12—C13—C14	119.28 (18)
C4A—C3A—H3A2	109.3	C15—C14—C13	120.63 (17)
C2—C3A—H3A2	109.3	C15—C14—H14	119.7
H3A1—C3A—H3A2	108	C13—C14—H14	119.7
C5—C4A—C3A	107.3 (8)	C14—C15—C10	121.24 (18)
C5—C4A—H4A1	110.3	C14—C15—H15	119.4
C3A—C4A—H4A1	110.3	C10—C15—H15	119.4
C5—C4A—H4A2	110.3	O2—C16—H16A	109.5
C3A—C4A—H4A2	110.3	O2—C16—H16B	109.5
H4A1—C4A—H4A2	108.5	H16A—C16—H16B	109.5
C4A—C5—C6	113.5 (4)	O2—C16—H16C	109.5
C6—C5—C4B	110.4 (3)	H16A—C16—H16C	109.5
C4A—C5—H5A	108.9	H16B—C16—H16C	109.5
C6—C5—H5A	108.9	C2—C3B—C4B	107.7 (7)
C4B—C5—H5A	131.7	C2—C3B—H3B1	110.2
C4A—C5—H5B	108.9	C4B—C3B—H3B1	110.2
C6—C5—H5B	108.9	C2—C3B—H3B2	110.2
C4B—C5—H5B	84.8	C4B—C3B—H3B2	110.2
H5A—C5—H5B	107.7	H3B1—C3B—H3B2	108.5
C4A—C5—H5C	128.3	C3B—C4B—C5	111.5 (7)
C6—C5—H5C	109.6	C3B—C4B—H4B1	109.3
C4B—C5—H5C	109.6	C5—C4B—H4B1	109.3
H5A—C5—H5C	81.9	C3B—C4B—H4B2	109.3
C4A—C5—H5D	83	C5—C4B—H4B2	109.3
C6—C5—H5D	109.6	H4B1—C4B—H4B2	108
			
C7—O1—N1—C9	0.36 (19)	C7—C8—C9—N1	1.2 (2)
C6—C1—C2—C3B	−19.4 (5)	C7—C8—C9—C10	−177.85 (16)
C7—C1—C2—C3B	160.8 (5)	N1—C9—C10—C11	166.10 (16)
C6—C1—C2—C3A	15.2 (5)	C8—C9—C10—C11	−14.9 (3)
C7—C1—C2—C3A	−164.6 (5)	N1—C9—C10—C15	−14.6 (3)
C1—C2—C3A—C4A	−48.6 (11)	C8—C9—C10—C15	164.40 (18)
C3B—C2—C3A—C4A	58.7 (9)	C15—C10—C11—C12	0.8 (3)
C2—C3A—C4A—C5	66.8 (13)	C9—C10—C11—C12	−179.88 (15)
C3A—C4A—C5—C6	−48.4 (12)	C10—C11—C12—C13	−0.5 (3)
C3A—C4A—C5—C4B	41.0 (10)	C16—O2—C13—C12	−3.3 (3)
C7—C1—C6—C5	−179.31 (18)	C16—O2—C13—C14	177.82 (17)
C2—C1—C6—C5	0.9 (3)	C11—C12—C13—O2	−179.06 (16)
C4A—C5—C6—C1	16.8 (6)	C11—C12—C13—C14	−0.2 (3)
C4B—C5—C6—C1	−13.1 (5)	O2—C13—C14—C15	179.53 (17)
N1—O1—C7—C8	0.39 (19)	C12—C13—C14—C15	0.6 (3)
N1—O1—C7—C1	−179.91 (14)	C13—C14—C15—C10	−0.3 (3)
C6—C1—C7—C8	−162.0 (2)	C11—C10—C15—C14	−0.4 (3)
C2—C1—C7—C8	17.8 (3)	C9—C10—C15—C14	−179.76 (17)
C6—C1—C7—O1	18.4 (2)	C1—C2—C3B—C4B	49.0 (10)
C2—C1—C7—O1	−161.79 (16)	C3A—C2—C3B—C4B	−36.8 (7)
O1—C7—C8—C9	−0.93 (19)	C2—C3B—C4B—C5	−61.8 (11)
C1—C7—C8—C9	179.44 (18)	C4A—C5—C4B—C3B	−57.8 (11)
O1—N1—C9—C8	−1.0 (2)	C6—C5—C4B—C3B	44.2 (10)
O1—N1—C9—C10	178.20 (14)		

### Hydrogen-bond geometry (Å, °)

**Table:**

*D*—H···*A*	*D*—H	H···*A*	*D*···*A*	*D*—H···*A*
C6—H6···O1	0.93	2.48	2.811 (3)	101
